# Response–Response Binding: New Evidence from Event-Related Potentials Data

**DOI:** 10.3390/brainsci14121183

**Published:** 2024-11-26

**Authors:** Biye Wang, Lu Wang, Tao Tao, Wei Guo

**Affiliations:** College of Physical Education, Yangzhou University, Yangzhou 225000, China; wangbiye@yzu.edu.cn (B.W.); mx120210489@stu.yzu.edu.cn (L.W.); mx120240506@stu.yzu.edu.cn (T.T.)

**Keywords:** response–response binding, action control, event-related potentials

## Abstract

Background: Response–response (RR) binding, involving the integration of independently planned and executed responses, presents a novel perspective on action control. While behavioral evidence on RR binding has been extensively examined, corresponding electrophysiological evidence remains scarce. This study aims to contribute novel insights into RR binding by event-related potentials (ERP) techniques to provide new evidence for RR binding. Methods: An adapted prime-probe paradigm was employed, in which the required responses could either involve repetition or change conditions from the prime to the probe phase. EEG data were collected from thirty-six participants, and ERP analysis focused on the peak amplitude and latency of the P1 and P3 components. Results: Notably shorter RTs were observed in the response–repetition conditions compared to others, consistent with previous findings on RR binding. Furthermore, the response–repetition conditions exhibited larger P3 amplitudes and shorter P1 and P3 latencies relative to other conditions. Conclusions: In summary, this study strengthens the evidence base for RR binding by bridging both behavioral and electrophysiological perspectives.

## 1. Introduction

Human actions are diverse, ranging from simple tasks such as pressing a button to complex activities like playing the piano. Researchers tended to explain action control by stimulus–response (SR) binding [1,2,3,4,5]. According to this perspective, responding to a stimulus results in the binding of stimulus and response features, integrating them into what is known as an event file. When stimulus features are repeated, the corresponding response features are retrieved, influencing subsequent actions—a phenomenon referred to as binding effects [2,6]. If the retrieved response aligns with the required response, it facilitates the action process; if it diverges, interference is introduced. For example, when responding to a red stimulus by pressing the “F” key and a green stimulus by pressing the “J” key, associations are formed between each stimulus and its corresponding key response. These SR bindings facilitate faster reactions when the same stimulus-response pair (e.g., pressing “F” for red or “J” for green) is repeated. However, if the required response changes, such as pressing a different key for the red stimulus or the green stimulus, reaction times are notably slower. The SR binding effect is highly robust and has received considerable attention in the field of action control [6].

In addition to SR binding, response–response (RR) binding also plays a significant role in action control. It has been proposed that RR binding operates similarly to SR binding, as both rely on the integration and retrieval of information [6]. However, RR binding differs from SR binding in that the event files involved in RR binding analysis encompass two (or more) independently planned and executed responses [7]. When two responses (R1 and R2) are integrated, a subsequent repetition of R1 triggers the retrieval of R2. If the retrieved response is consistent with the required response, it facilitates the action (e.g., reducing response time); if it is inconsistent, it interferes with the response (e.g., increasing response time). For example, imagine playing a piece on the piano: Pressing one key (R1) might become bound to the subsequent keypress (R2), enhancing the fluidity of the performance. However, if the second key (R2) unexpected changes, this disrupts the sequence, requiring additional effort and practice to adjust. To explore the intricacies of RR binding, Moeller and Frings [8] made significant modifications to the prime-probe paradigm, which was previously used to investigate SR binding [9]. This paradigm was designed to involve two individually planned responses to Task A and Task B during both the prime and probe phases. Task A primarily involved the classification of stimulus shapes, while Task B focused on the classification of stimulus colors. Responses in Task A and/or Task B were referred to as “repetition” when they remained consistent from the prime to the probe phase and as “change” when they differed. Within this experimental framework, responses could be executed using a single effector, such as the hands, or across multiple effectors, such as responding to Task A with the hands and to Task B with the foot [10]. Additionally, stimuli varied not only in shape and color but also included numbers or uppercase letters, with participants responding using distinct finger-operated keys [11].

Several factors have been explored for their influence on RR binding effects, including nonroutine action sequences, task switches, temporal order information, response–stimulus intervals (RSI), and different effectors. Nonroutine action sequences have the potential to bind noncontiguous responses, and the magnitude of these bindings is comparable to those observed in contiguous responses [12]. Moreover, task-switching studies have revealed that binding is not limited to individually planned responses; it can extend to responses triggered by task-switching [8]. The influence of temporal order information on RR binding was scrutinized by manipulating sequence order. Results indicated that the binding effect persists independent of alterations in sequence order. Consequently, RR binding does not encode temporal order information [11]. Further exploration of temporal dynamics examined the influence of RSI on RR binding, demonstrating that binding can occur not only between independently planned responses but also remain effective for at least 6 s [13]. Additionally, research has shown that RR binding can happen across different effectors, though the effect is weaker when using multiple effectors compared to a single effector [10].

The aforementioned behavioral studies have indeed enriched our understanding of RR binding. However, neuroimaging evidence provides additional insights into this process. Geissler, Moeller, and Frings extended this research by identifying the neural mechanisms underlying RR binding using functional near-infrared spectroscopy (fNIRS). Their work revealed specific activation patterns in the middle and superior frontal regions: the anterior dorsolateral prefrontal cortex was associated with the response repetition condition, while the right anterior dorsolateral prefrontal cortex was linked with the response–change condition [14]. A particularly noteworthy contribution of their research is the observation that RR binding maintains its coherence most effectively during the initial six seconds following integration. Nevertheless, this study was constrained by the low temporal resolution of fNIRS, which limited its focus to the retention phase of RR binding, without addressing other critical aspects of the process. In contrast, ERP technology, known for its high temporal resolution [15], may be more suitable for directly investigating RR binding. For instance, previous research has used ERP to study binding mechanisms in action control [16,17], and although it did not specifically target RR binding, it clearly demonstrated the feasibility of employing ERP in the investigation of RR binding. The P1 component is characterized by an early positive deflection in EEG activity occurring within a 60–130 ms time window after stimulus presentation, associated with the automatic processing of stimuli [18]. As an early component, P1 operates independently of conscious control and completes binding automatically [19]. The P3 component, on the other hand, is a late positive deflection occurring within a 300–700 ms window post-stimulus, related to response preparation during action execution [20,21,22]. When stimulus presentation and response execution occur in close temporal proximity, SR binding happens automatically [23]. We hypothesize that within a prime-probe paradigm, the previously observed RR binding effect will be replicated, as reflected in the response time patterns. Additionally, the P1 and P3 components are expected to show shorter latencies, with the P3 component exhibiting greater amplitude under response repetition conditions.

The present study utilizes ERP techniques, known for their remarkable temporal precision, within a modified RR binding paradigm [11]. The primary objective of this investigation is to provide new evidence for response–response (RR) binding through the analysis of ERP. In terms of behavioral outcomes, the study anticipates the replication of findings observed in prior RR binding experiments. Moreover, from an electrophysiological perspective, the anticipation is to identify a distinct neural signature of the binding effect in the ERP components.

## 2. Materials and Methods

### 2.1. Participants

The effect sizes (η_p_^2^) of RR binding effects in previous experiments were found to be substantial, surpassing a value of 0.25 (Moeller and Frings, 2019a: η_p_^2^ = 0.29 and η_p_^2^ = 0.31; Moeller and Frings, 2019b: η_p_^2^ = 0.54; and Moeller and Frings, 2019c: η_p_^2^ = 0.37 and η_p_^2^ = 0.55). In light of this, the present study aimed to detect an effect size of at least η_p_² = 0.25, assuming a significance level (α) of 0.05 and a statistical power (1-β) of 0.8. Conducting a power analysis utilizing the G*Power software 3.1 indicated that a minimum of 24 participants would be necessary [24]. For the current study, a total of thirty-six undergraduate students (13 female, 23 male) participated in this experiment (median age = 21 years, range = 19–24). All participants were right-handed and had normal or corrected-to-normal vision. They had no history of any neurological or psychiatric disorders. Written informed consent was obtained from each participant prior to the experiment. As an acknowledgment of their involvement, participants received partial course credit upon completion of the experiment.

### 2.2. Materials

The experimental program was implemented using the Matlab Psychtoolbox-3.0, a widely recognized psychological toolbox. The experiment was prepared and completed in the EEG testing room. Participants completed the experimental task using a standard computer keyboard. Participants sat comfortably in a quiet and dimly lit room, and the stimuli were presented on a computer monitor (19 inches) located 60 cm from the participant. Instructions and target stimuli were displayed on the computer screen in white, represented by an RGB value of 255, set against a black background characterized by an RGB value of 0. The screen resolution was configured to 1980 × 1080 pixels. Stimuli were the letters A, B, C, and D and the digits 1, 2, 3, and 4. All letters and digits subtended a horizontal visual angle of 0.4° to 0.6°, with a corresponding vertical visual angle of 0.5°. To provide responses, participants were required to interact with a computer keyboard, employing one of four designated keys.

### 2.3. Procedure

Participants were individually tested within a quiet and dimly lit room, with instructions presented on the screen. During the task, participants positioned their middle and index fingers on the Q, E, U, and O keys of a standard computer keyboard. The objective consistently entailed pressing the designated key corresponding to the presented letter or digit. For instance, when stimulus A/1 appeared, participants were required to press the Q key using their left middle finger. Similarly, upon presentation of stimulus B/2, the left index finger was to be used to press the E key. Response to stimulus C/3 necessitated the use of the right index finger to press the U key, while stimulus D/4 elicited a response involving the right middle finger pressing the O key. All stimuli were centrally presented on the screen throughout the task duration.

**Figure 1:**
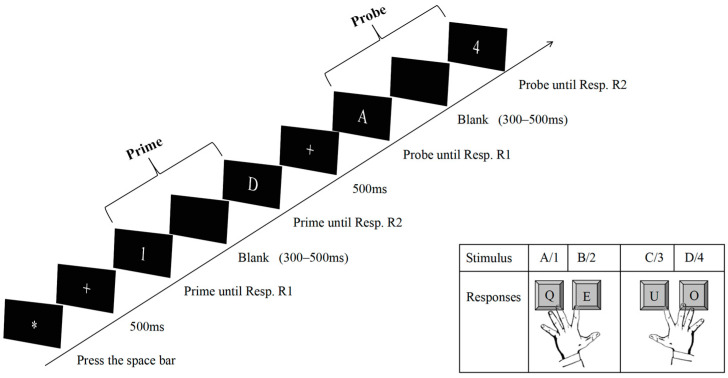
Schematic example for the sequence of events in one trial. Participants gave two successive responses, to R1 and R2, both in the prime and in the probe. They positioned their middle and index fingers on the Q, E, U, and O keys of a standard computer keyboard, while associating stimulus A/1 with the Q key, stimulus B/2 with the E key, stimulus C/3 with the U key, and stimulus D/4 with the O key. The depicted trial is an example of the R1rR2r condition: response repetition is required for both R1 and R2. Stimuli are not drawn to scale.

At the onset of each trial, an asterisk was centrally presented on the screen, signifying to the participant that the initiation of the trial required pressing the space bar. Subsequently, the asterisk was replaced by a plus sign, which persisted for a duration of 500 ms. Following this, the first prime letter or digit appeared in the center of the screen until the participant pressed one of the four keys, indicating the prime response R1. The stimulus disappeared when the participant responded, and the second prime letter or digit stimulus was presented after a blank screen for 200–500 ms (RSI) until the participant responded, indicating the prime response R2. A fixation mark appeared for 500 ms and was followed by the first probe letter or digit until the participant responded, indicating the probe response R1. Then, the second probe letter or digit was presented after a blank screen for 200–500 ms (RSI) until the participant responded, indicating the probe response R2. Finally, an asterisk presented in the center of the screen indicated that the participant could start the next trial. Participants performed two separate responses (response R1 and response R2) in each prime and probe phase. Participants could only perform response R2 after performing response R1 because of the sequential nature of events in each trial. In the case of an incorrect response R1 or R2 from prime to probe, a red exclamation point appeared for 1500 ms to remind the participant to respond as quickly as possible but without errors. At the end of each block, participants were prompted to take a short break, after which they continued the task at their own pace (Figure 1).

The relation of response R1 from prime to probe (repetition vs. change) was varied orthogonally to the relation of response R2 from prime to probe (repetition vs. change), while target stimuli did not repeat in the same trial. In R1 repetition trials (R1r), the same response was required to the stimulus indicating prime response R1 and the one indicating probe response R1. In R1 change trials (R1c), different responses were required to the stimulus indicating prime response R1 and the one indicating probe response R1. In R2 repetition trials (R2r), the same response was required to the stimulus indicating prime response R2 and the one indicating probe response R2. In R2 change trials (R2c), different responses were required to the stimulus indicating prime response R2 and the one indicating probe response R2. These relations resulted in the four conditions R1rR2r, R1rR2c, R1cR2r, and R1cR2c, which are necessary to measure RR binding effects (Table 1). The experimental block consisted of 256 experimental trials. Before the experimental block started, participants practiced 32 trials to familiarize themselves with the keys and task.

### 2.4. Study Design

The experiment employed a 2 × 2 within-subjects design, consisting of two within-subject factors: response R1 relation (response repetition vs. response change from prime to probe) and response R2 relation (response repetition vs. response change from prime to probe).

### 2.5. Electrophysiological Analysis

#### 2.5.1. EEG Recording

Each electroencephalogram (EEG) and electrooculogram (EOG) were amplified by the SynAmps2 system. Electrophysiological data were recorded from 64 scalp sites via tin electrodes and positioned according to the International 10–20 electrode system. Electrode-skin impedances were kept below 5 KΩ for all electrodes. The raw EEG data were referenced online to a reference electrode located between Cz and CPz, with the ground electrode placed at AFz. EOG data were recorded from four additional bipolar channels. Vertical electrooculography (VEOG) signals were recorded with electrodes positioned 1 cm above and below the midpoint of the left eye’s orbit, while horizontal electrooculography (HEOG) signals were recorded using electrodes placed at the outer canthi of the left and right eyes. All signals were sampled at 500 Hz and band-pass filtered within a 0.05–100 Hz frequency range. The behavioral data were recorded synchronously when EEG signals were collected.

#### 2.5.2. EEG Preprocessing

EEG data were preprocessed and analyzed using the EEGLAB v2022.1 [25] and ERPLAB v9.20 [26] toolkits in MATLAB R2020b. All electrode recordings were re-referenced offline relative to a mean value placed on the left and right mastoid (M1, M2). The sampling rate was reduced to 250 Hz per channel for offline analysis to facilitate analysis of the data. A band-pass filter was applied, starting with a high-pass filter at 0.1 Hz and followed by a low-pass filter at 20 Hz (24 dB/oct). Artifact rejection criteria were set at ±70 μv. EEG epochs of 2000 ms were extracted offline, stimulus-locked to the probe. Only trials with correct responses to both the prime and the probe were included. Correct trials were segmented, superimposed, and averaged for each condition. Finally, the averages of individual conditions were calculated separately for all participants.

#### 2.5.3. ERP Analyses

ERP components were identified based on visual inspection and previous ERP waveform studies [27,28,29]. The time window spanned from 100 ms before R2 stimulus presentation to 700 ms after stimulus onset, with the 100 ms preceding stimulus presentation used for baseline correction. P1 (70–150 ms) and P3 (300–500 ms) were selected as time-windows for analysis. For P1, the focus was on the occipital electrode Oz [30]. For P3, three midline electrodes (Fz, Cz, and Pz) were examined [18]. Peak amplitudes and latency were measured within the respective time windows at the corresponding electrodes.

## 3. Results

### 3.1. Behavioral Results

**Figure 2:**
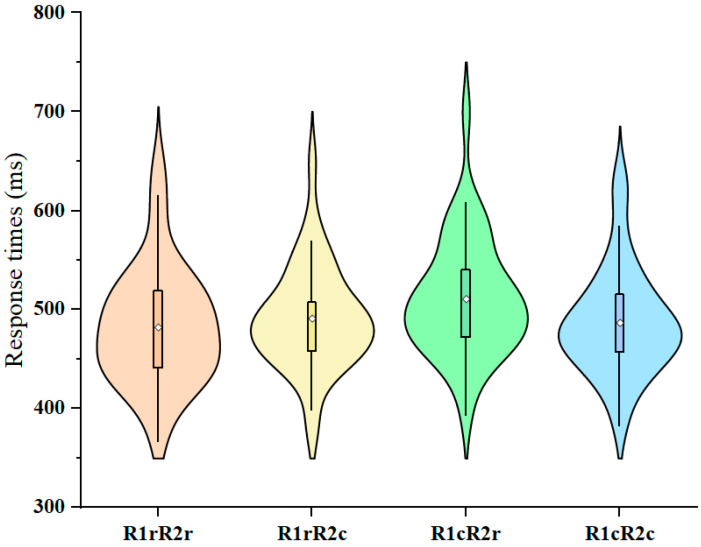
Violin plots of response times (ms) data under the four conditions (R1rR2r, R1rR2c, R1cR2r, and R1cR2c). Orange indicates R1rR2r, yellow indicates R1rR2c, green indicates R1cR2r, and blue indicates R1cR2c. The hollow rhombus inside the rectangle represents the mean value.

We reported the response times and error rates under four conditions (Figure 2). Prime R1 and prime R2 were integrated, and repeating either of them as probe R1 may trigger retrieval of the other, which affects the performance of probe R2. If the retrieved response is consistent with the currently required one, responding is facilitated. If the retrieved response is inconsistent with the currently required one, responding is impaired. Thus, the dependent variable of interest for the RR binding effect was the performance of probe R2. We considered that only trials with correct responses R1 and R2 in prime and probe should be included in the analysis of response times (RTs). The error rate (ERs) for prime responses was 3.9%. The probe error rates were 0.8% for R1 and 2.3% for R2. RTs that were more than 1.5 interquartile ranges above the third quartile of the participant’s RT distribution [24], and RTs that were shorter than 200 ms were excluded from the analysis. Due to these constraints, 12.9% of the trials were excluded from the RT analyses. For mean RTs and ERs, see Table 2.

In a 2 (R1 relation: repetition vs. change) × 2 (R2 relation: repetition vs. change) multivariate analysis of variance (MANOVA) on probe response R2 RTs with Pillai’s trace as the criterion, the main effect of the R1 relation was significant: F (1, 35) = 42.220, *p* < 0.001, and η_p_^2^ = 0.547. Participants responded faster if R1 was repeated (M = 487 ms and SD = 53 ms) than if it had to be changed (M = 499 ms and SD = 55 ms) from prime to probe. The main effect of the R2 relation was not significant: F (1, 35) = 3.342, *p* = 0.076, and η_p_^2^ = 0.087. More importantly, the interaction of the R1 and R2 relations was significant as well: F (1, 35) = 28.432, *p* < 0.001, and η_p_^2^ = 0.448, indicating binding between the responses. Follow-up analyses specified that repeating R1 facilitated performance if R2 was repeated from prime to probe, and changing R1 facilitated performance if R2 was changed from prime to probe, as well. In the same analysis on error rates, the main effect of the R1 relation was not significant: F (1, 35) = 1.853, *p* = 0.182, and η_p_^2^ = 0.050. The main effect of the R2 relation was not significant: F (1, 35) = 0.465, *p* = 0.500, and η_p_^2^ = 0.013. The interaction of the R1 relation and the R2 relation was not significant: F (1, 35) = 3.623, *p* = 0.065, and η_p_^2^ = 0.094.

### 3.2. ERP Results

#### 3.2.1. P1 Component (70–150 ms)

##### Amplitude

**Figure 3:**
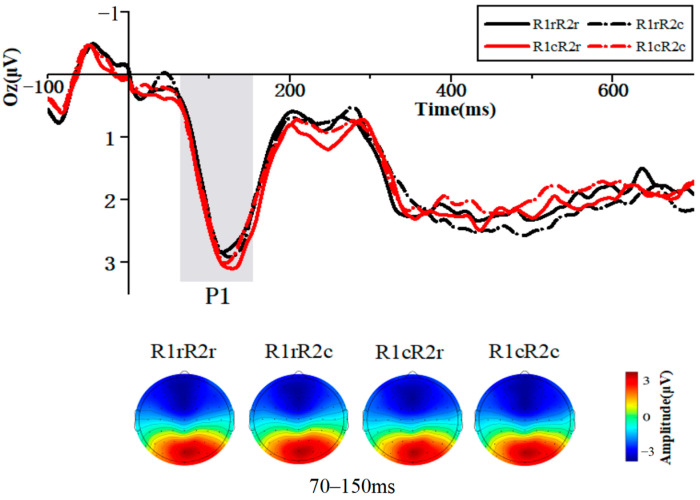
Grand average waveforms of the ERP under the four conditions. The time window of the P1 is highlighted. These waveforms represent the mean values of the data at the electrode site Oz. The corresponding scalp topographies for every condition are provided below. Mean amplitude between 70 ms and 150 ms was used to plot scalp maps.

The repeated measures ANOVA of the P1 component at electrode site Oz revealed the main effect of R1 was not significant, F (1, 35) = 0.763, *p* = 0 0.388, and η_p_^2^ = 0.021. The main effect of R2 was not significant: F (1, 35) = 0.755, *p* = 0.391, and η_p_^2^ = 0.021. The interaction of the R1 and R2 relations was not significant: F (1, 35) = 0.473, *p* = 0.496, and η_p_^2^ = 0.013 (Figure 3).

##### Latency

At electrode site Oz, the main effect of R1 was not significant: F (1, 35) = 0.255, *p* = 0.617, and η_p_^2^ = 0.007. The main effect of R2 was not significant: F (1, 35) = 1.307, *p* = 0.261, and η_p_^2^ = 0.036. However, the interaction of the R1 and R2 relations was significant: F (1, 35) = 4.695, *p* = 0.037, and η_p_^2^ = 0.118, indicating binding between the responses. The P1 latency induced by R2 repeated (M = 118.444 ms and SD = 17.586 ms) was shorter than that induced by the changed (M = 123.444 ms and SD = 17.028 ms) only if R1 was repeated.

#### 3.2.2. P3 Component (300–500 ms)

##### Amplitude

**Figure 4:**
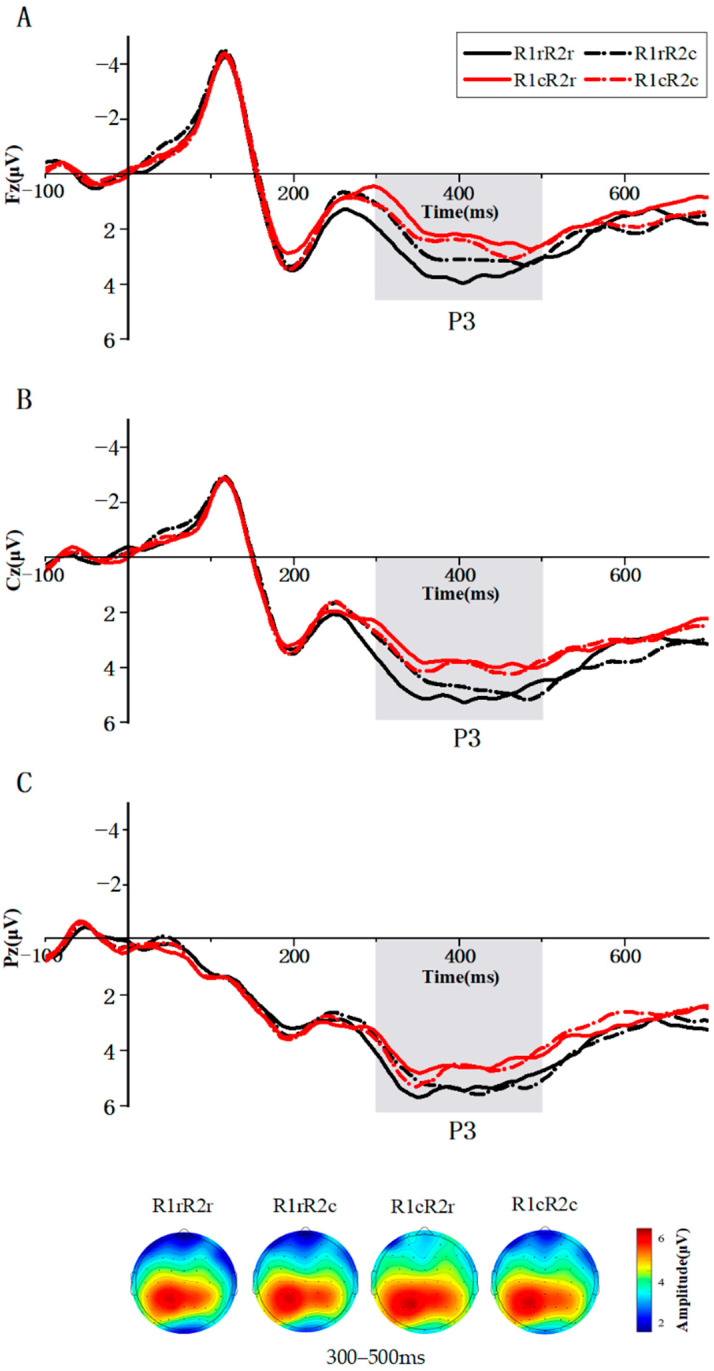
Grand average waveforms of the ERP under the four conditions (R1rR2r, R1rR2c, R1cR2r, and R1cR2c). The time window of P3 is highlighted. (**A**) These waveforms represent the mean values of the data at the electrode site Fz. (**B**) These waveforms represent the mean values of the data at the electrode site Cz. (**C**) These waveforms represent the mean values of the data at the electrode site Pz. The corresponding scalp topographies for every condition are provided below. Mean amplitude between 300 ms and 500 ms was used to plot scalp maps.

The repeated measures ANOVA of the P3 component at electrode site Fz revealed the main effect of the R1 relation was significant: F (1, 35) = 9.076, *p* = 0.005, and η_p_^2^ = 0.206. The P3 peak amplitude induced by R1 repeated (M = 5.549 μv and SD = 4.440 μv) was larger than that induced by the changed (M = 4.337 μv and SD = 3.606 μv) from prime to probe. The main effect of R2 was not significant: F (1, 35) = 0.751, *p* = 0.392, and η_p_^2^ = 0.021. The interaction of the R1 and R2 relations was significant: F (1, 35) = 4.446, *p* = 0.042, and η_p_^2^ = 0.113. The P3 peak amplitude induced by R2 repeated (M = 5.926 μv and SD = 4.550 μv) was larger than that induced by the changed (M = 5.172 μv and SD = 4.567 μv) if R1 was repeated (t = 2.153, *p* = 0.038). The P3 peak amplitude induced by R1 repeated (M = 5.926 μv and SD = 4.550 μv) was larger than that induced by the changed (M = 4.206 μv and SD = 3.912 μv) if R2 was repeated (t = 3.755 and *p* = 0.001) (Figure 4A).

The repeated measures ANOVA of the P3 component at electrode site Cz revealed the main effect of the R1 relation was significant: F (1, 35) = 6.387, *p* = 0.016, and η_p_^2^ = 0.154. The P3 peak amplitude induced by R1 repeated (M = 6.923 μv and SD = 5.292 μv) was larger than that induced by the changed (M = 5.757 μv and SD = 4.116 μv) from prime to probe. The main effect of the R2 relation was not significant: F (1, 35) = 0.289, *p* = 0.594, and η_p_^2^ = 0.008. The interaction of the R1 and R2 relations was not significant: F (1, 35) = 1.680, *p* = 0.203, and η_p_^2^ = 0.046 (Figure 4B).

The repeated measures ANOVA of the P3 component at electrode site Pz revealed the main effect of the R1 relation was significant: F (1, 35) = 4.406, *p* = 0.043, and η_p_^2^ = 0.112. The P3 peak amplitude induced by R1 repeated (M = 6.997 μv and SD = 3.114 μv) was larger than that induced by the changed (M = 6.397 μv and SD = 2.904 μv) from prime to probe. The main effect of the R2 relation was not significant: F (1, 35) = 0.157, *p* = 0.694, and η_p_^2^ = 0.004. The interaction of the R1 and R2 relations was significant: F (1, 35) = 8.232, *p* = 0.007, and η_p_^2^ = 0.190. The P3 peak amplitude induced by R2 repeated (M = 7.333 μv and SD = 3.503 μv) was larger than that induced by the changed (M = 6.662 μv and SD = 2.968 μv) if R1 was repeated (t = 2.433 and *p* = 0.020). The P3 peak amplitude induced by R1 repeated (M = 7.333 μv and SD = 3.503 μv) was larger than that induced by the changed (M = 6.144 μv and SD = 2.872 μv) if R2 was repeated (t = 3.870 and *p* < 0.001) (Figure 4C).

##### Latency

At electrode site Fz, both main effects were significant: F (1, 35) = 17.862, *p* < 0.001, and η_p_^2^ = 0.338 for the R1 relation and F (1, 35) = 12.325, *p* = 0.001, and η_p_^2^ = 0.260 for the R2 relation. More importantly, the interaction between the R1 relation and the R2 relation was significant as well: F (1, 35) = 15.656, *p* < 0.001, and η_p_^2^ = 0.309. The P3 latency induced by R2 repeated (M = 384.361 ms and SD = 44.122 ms) was shorter than that induced by the changed (M = 425.889 ms and SD = 52.915 ms) if R1 was repeated. The P3 latency induced by R1 repeated (M = 384.361 ms and SD = 44.122 ms) was shorter than that induced by the changed (M = 426.500 ms and SD = 59.072 ms) if R2 was repeated.

At electrode site Cz, both main effects were significant: F (1, 35) = 12.675, *p* = 0.001, and η_p_^2^ = 0.266 for the R1 relation and F (1, 35) = 4.146, *p* = 0.049, and η_p_^2^ = 0.106 for the R2 relation. More importantly, the interaction between the R1 relation and the R2 relation was significant as well: F (1, 35) = 23.558, *p* < 0.001, and η_p_^2^ = 0.402. The P3 latency induced by R2 repeated (M = 394.417 ms and SD = 50.235 ms) was shorter than that induced by the changed (M = 425.000 ms and SD = 55.235 ms) if R1 was repeated. The P3 latency induced by R1 repeated (M = 394.417 ms and SD = 50.235 ms) was shorter than that induced by the changed (M = 436.167 ms and SD = 59.600 ms) if R2 was repeated.

At electrode site Pz, both main effects were significant: F (1, 35) = 4.166, *p* = 0.049, and η_p_^2^ = 0.106 for the R1 relation and F (1, 35) = 5.454, *p* = 0.025, and η_p_^2^ = 0.135 for the R2 relation. More importantly, the interaction between the R1 relation and the R2 relation was significant as well: F (1, 35) = 33.700, *p* < 0.001, and η_p_^2^ = 0.491. The P3 latency induced by R2 repeated (M = 382.361 ms and SD = 56.567 ms) was shorter than that induced by the changed (M = 421.667 ms and SD = 48.329 ms) if R1 was repeated. The P3 latency induced by R1 repeated (M = 382.361 ms and SD = 56.567 ms) was shorter than that induced by the changed (M = 402.389 ms and SD = 65.613 ms) if R2 was repeated.

## 4. Discussion

In the present study, we investigated the behavioral and electrophysiological characteristics of RR binding in an adapted prime-probe paradigm [8], employing ERP as a key methodological tool. This approach facilitated the discovery of new evidence for RR binding. The effects of RR binding were prominently evident in the behavioral outcomes, particularly in response times (RTs). Specifically, RTs were shorter when all responses were repeated or changed compared to when only one response was repeated. This finding corroborates the RR binding effects initially proposed by Moeller and Frings (2019) [8]. Furthermore, both the interference and facilitation effects of binding [14] were observed in the present study. The interference effect, also known as partial repetition costs, refers to the phenomenon that the partial repetition of content in an event file usually produces interference on subsequent responses, resulting in increased RTs or error rates [31]. Conversely, the facilitation effect, also known as the repetition benefit, occurs when the complete repetition of content in an event file facilitates subsequent responses, resulting in decreased RTs or error rates [32]. Importantly, this study not only confirmed the existence of RR binding effects but also provided support for the theoretical frameworks of Theory of Event Coding (TEC) and Binding and Retrieval in Action Control (BRAC). These frameworks propose that facilitation occurs when the retrieved response aligns with the currently required response, whereas interference arises in cases of incongruence [6]. Encouragingly, these predicted effects were indeed observable within the present study’s findings. A notable observation was the absence of RR binding effects in terms of error rates, likely due to the participants’ exceptionally high response accuracy with a mean accuracy of 97.93%. This suggests that the high accuracy levels may have masked potential differences in error rates related to RR binding.

An important finding emerged from the analysis, revealing a significant interaction between the relationships of R1 and R2 on the amplitude of the P3 component, as evidenced at electrode sites Fz and Pz. Specifically, a distinctive pattern emerged wherein greater amplitude in P3 potentials was manifest in instances of R2 repetition compared to R2 change, particularly within the context of R1 repetition. This discernible interaction closely aligns with patterns observed in the behavioral outcomes reported in the prior study [8]. This alignment suggests a potential link to the integration between responses. It is noteworthy that the experimental design, in this case, allowed each response to correspond to two distinct categories of stimuli. As a result, it is unlikely that the binding phenomenon observed could be attributed to the stimuli themselves; rather, the binding effects seem to stem from the unique response relations. Speculatively, the observed increase in P3 amplitude might be attributed to the intricate integration process underlying multiple responses. Furthermore, the interaction arising from the interplay of R1 and R2 relationships was notably evident at electrode sites Fz and Pz. When R2 repetition was enacted, it signaled a process of retrieval, indicating a retrieval-execution connection. Conversely, in instances of R2 change, a distinct process was at play, where response planning and execution occurred independently. This dichotomy can be perceived as a transformation of cognitive rules or strategies, a phenomenon well-established in the cognitive neuroscience literature [20,33,34,35]. This pertinent insight aids in elucidating the observed interaction at electrode site Fz, as it lends support to the interpretation that the interference effect can be conceptualized as a manifestation of the transformation of cognitive rules, particularly at the neural level associated with electrode site Fz.

The EEG outcomes of the present study exhibit a significant alignment with the conclusions drawn from fNIRS investigations, thereby reinforcing the pivotal role attributed to the frontal cortex in the process of RR binding. Prior research has underscored the connection between P3 amplitudes and the activation of the inferior parietal region in the context of stimulus-response (SR) binding [28]. This congruence substantiates the rationale behind the observed interaction at electrode site Pz, suggesting that RR binding may, in part, engage the same neural region as SR binding—namely, the parietal region. Furthermore, an intriguing outcome emerged, revealing a significant interaction between the relationships of R1 and R2 on the latency of the P3 component, localized at electrode sites Fz, Cz, and Pz. Notably, when R1 changed, no significant differences were observed irrespective of R2’s relations. However, in the scenario of R1 repetition, a notable pattern emerged: the P3 latency elicited by a repeated R2 response was notably shorter compared to the latency induced by a changed R2 response. This latency variation is hypothesized to be associated with the intricate process of response preparation during response execution [36,37]. It is conceivable that this alteration in latency could potentially stem from the pre-activation of responses during the preparatory phase. In summation, the empirical findings of this study offer robust electrophysiological evidence supporting the presence of RR binding effects. The P3 amplitude signifies that RR binding may coincide with the activation of either parietal or frontal regions. Moreover, the temporal dynamics of RR binding effects are suggested to be influenced by the pre-activation of responses during the retrieval phase.

An additional notable finding is the significant interaction discovered between the relationships of R1 and R2 on the latency of the P1 component, as observed at electrode site Oz. Notably, when R1 changed, no significant differences were observed irrespective of R2’s relations. However, in instances of R1 repetition, a distinct pattern emerged: the P1 latency elicited by a repeated R2 response was notably shorter compared to the latency induced by a changed R2 response. The Theory of Event Coding (TEC) offers a plausible explanation for this observed pattern. As proposed by Hommel, the execution of an action triggers a state of pre-activation for the stimulus corresponding to that specific response [3]. In light of this, it is reasonable to speculate that the observed shortening of P1 latency may be attributed to this pre-activation phenomenon. Specifically, the acceleration of visual information processing due to the pre-activation of the stimulus corresponding to the response.

In summary, this study provides novel ERP-based evidence supporting the existence of RR binding effects. The observed P3 amplitudes suggest that RR binding may engage the frontal and parietal regions, while the P1 latency results indicate that RR binding influences early visual processing through pre-activation mechanisms. These findings represent an important step toward understanding the neural substrates of RR binding. However, further research is necessary to fully explore the scope and implications of this phenomenon. Particularly, further investigation should aim to clarify the similarities and differences between SR and RR binding, as well as their interactions when they occur simultaneously.

## 5. Conclusions

The present study offers robust evidence of response–response (RR) binding effects, integrating findings from both behavioral and electrophysiological domains. Behaviorally, consistently repeated or changed responses yielded shorter response times (RTs) compared to singular repetitions. Electrophysiological analysis further revealed increased P3 amplitudes and reduced latencies when the retrieved response matched the expected response, indicating potential pre-activation effects. Moreover, shorter P1 latencies were observed under these conditions, suggesting a link between RR binding and the preparation for visual information processing.

In conclusion, the convergence of behavioral and electrophysiological findings strengthens the robustness of RR binding effects. The interplay between behavioral response patterns and neurophysiological markers offers insights into the intricate mechanisms underlying response integration and retrieval, thereby enriching our comprehension of the cognitive substrates underpinning RR binding.

## Figures and Tables

**Table 1:** Examples of stimuli presented to indicate prime and probe responses R1 and R2 for all four conditions.

R1 Relation	R2 Relation		Prime Stimuli	Probe Stimuli
Repetition	Repetition	(R1rR2r)	4→C	D→3
	Change	(R1rR2c)	4→B	D→3
Change	Repetition	(R1cR2r)	A→C	D→3
	Change	(R1cR2c)	A→B	D→3

**Table 2:** Mean response times (RTs, in milliseconds) and mean error rates (ERs, in percentages), along with the amplitudes (in μV) and latencies (in milliseconds) of the P1 and P3 components for probe responses (R2), are presented as a function of the response R1 relation and response R2 relation.

	Probe R2		R2 Repetition	R2 Change
Behavioral	RT	R1 change	511 (9.8)	487 (9.0)
		R1 repetition	482 (9.9)	491 (8.7)
		Priming effect	29 (−0.1)	−4 (0.3)
	ER	R1 change	8.3 (0.8)	7.4 (0.6)
		R1 repetition	7.8 (0.7)	9.5 (0.9)
		Priming effect	0.5 (0.1)	−2.1 (−0.3)
P1 (Oz)	Amplitude	R1 change	3.6 (2.6)	3.6 (2.7)
		R1 repetition	3.5 (2.6)	3.5 (2.3)
	Latency	R1 change	120.2 (17.2)	118.8 (15.7)
		R1 repetition	118.4 (17.6)	123.4 (17.0)
P3 (Fz)	Amplitude	R1 change	4.2 (3.9)	4.5 (3.7)
		R1 repetition	5.9 (4.6)	5.2 (4.6)
	Latency	R1 change	426.5 (59.1)	421.7 (63.2)
		R1 repetition	384.4 (44.1)	425.9 (53.0)
P3 (Cz)	Amplitude	R1 change	5.6 (4.2)	6.0 (4.2)
		R1 repetition	7.0 (5.2)	6.9 (5.5)
	Latency	R1 change	436.2 (59.6)	420.9 (59.2)
		R1 repetition	394.4 (50.2)	425.0 (55.2)
P3 (Pz)	Amplitude	R1 change	6.1 (2.9)	6.7 (3.2)
		R1 repetition	7.3 (3.5)	6.7 (3.0)
	Latency	R1 change	402.4 (65.6)	382.0 (56.3)
		R1 repetition	382.4 (56.6)	421.7 (48.3)

Note. Standard errors of the means are in parentheses.

## Data Availability

The original contributions presented in the study are included in the article; further inquiries can be directed to the corresponding authors. Due to privacy and ethical restrictions, the data are not publicly available.
